# Factors Associated with Pre-Vaccination SARS-CoV-2 Infection Risk among Hospital Nurses Facing COVID-19 Outbreak

**DOI:** 10.3390/ijerph182413053

**Published:** 2021-12-10

**Authors:** Luca Coppeta, Cristiana Ferrari, Andrea Mazza, Marco Trabucco Aurilio, Stefano Rizza

**Affiliations:** 1 Department of Occupational Medicine, University of Rome Tor Vergata, 00133 Rome, Italy; lcoppeta@gmail.com (L.C.); cristianaferrari.md@gmail.com (C.F.); andreamazza92@libero.it (A.M.); 2 Department of Medicine and Health Sciences “V. Tiberio”, University of Molise, 86100 Campobasso, Italy; marco.trabuccoaurilio@unimol.it; 3 Department of Systems Medicine, University of Rome Tor Vergata, 00133 Rome, Italy

**Keywords:** SARS-CoV-2, health care workers, COVID-19 outbreak, night shift

## Abstract

The objective of this work was to evaluate the magnitude of COVID-19 spread and the related risk factors among hospital nurses employed in a COVID hospital in Rome, before the beginning of the vaccination programmes commenced in 2021. Participants periodically underwent (every 15–30 days) nasopharyngeal swab and/or blood sample for SARS-CoV-2 IgG examination. From 1 March 2020 to 31 December 2020, we found 162 cases of COVID-19 infection (*n* = 143 nasopharyngeal swab and *n* = 19 IgG-positive) in a total of 918 hospital nurses (17.6%). Most SARS-CoV-2-infected hospital nurses were night shift workers (NSWs), smokers, with higher BMI and lower mean age than that of individuals who tested negative. After adjusting for covariates, age (OR = 0.923, 95% C.I. 0.895–0.952), night shift work (OR = 2.056, 95% C.I. 1.320–2.300), smoking status (OR = 1.603, 95% C.I. 1.080–2.378) and working in high-risk settings (OR = 1.607, 95% C.I. 1.036–2.593) were significantly associated with SARS-CoV-2 hospital infection, whereas BMI was not significantly related. In conclusion, we found a high prevalence of SARS-CoV-2 infection among hospital nurses at a Rome COVID hospital in the pre-vaccination period. Smoking, young age, night shift work and high-risk hospital settings are relevant risk factors for hospital SARS-CoV-2 infection; therefore, a close health surveillance should be necessary among hospital nurses exposed to SARS-CoV-2.

## 1. Introduction

Although the severe acute respiratory syndrome coronavirus 2 (SARS-CoV-2) pandemic continues to spread worldwide, health systems deal with the issue of how to prevent the contagion of healthcare workers (HCWs). Since the first emergency of the SARS-CoV-2 pandemic in December 2019, HCWs have been identified at high risk to be infected and to become a vehicle of contagion for their patients, colleagues, family members and external contacts [1]. Data from the World Health Organization (WHO) reports that 14% of COVID-19 cases happened among health workers, despite them representing less than 3% of the population in most countries [2]; however, availability and quality of those studies are limited.

In recent studies, the risk for frontline healthcare operators was found to be particularly higher than the general population [3,4,5]. Analysing data from 25 studies with a large sample size (over 1000 participants), a recent systematic review found a pooled SARS-CoV-2 seroprevalence rate of 8% in HCWs [6]. However, due to the wide range of infection control measures adopted in different health care settings and national procedures, along with individual behaviours of operators [7], the rate of SARS-CoV-2-infected HCWs varies in different countries.

Italy was in the group of countries heavily hit by the SARS-CoV-2 spread at the very beginning of the pandemic; from February 2020 to the present date, over 4.5 million cases have occurred in the Italian region, 140,000 of which involved HCWs [8]. Epidemiological studies reported different rate of infection among Italian HCWs in different periods and across regions, ranging from 6.9 to 28.5% [9,10,11]. From March 2020 to May 2020, we reported a first wave hospital prevalence rate of 2.5% for SARS-CoV-2 infection and the risk appeared related to obesity (BMI > 30) and night shift work, but not with hospital working area [12]. However, aggregate data on a labor surveillance survey on 10,654 health operators in six Italian hospitals, collected during March and April 2020, revealed that the rate of SARS-CoV2 infection varied between regions from 3.0 to 22.0%, but it is not related to that kind of hospital setting [13]. Therefore, detection of COVID-19 infection prevalence among different risk groups of HCWs may help in rating the SARS-CoV-2 diffusion in the healthcare setting and evaluating the efficacy of taken measures in order to protect operators and susceptible patients [14]. However, it remains unclear whether, compared with HCWs with medium to low risks of COVID-19 infection, those with high risk have a higher rate of positive individuals.

In this study, we performed a retrospective investigation of SARS-CoV-2 infection rate in a large cohort of nurses employed in a Rome COVID hospital (Tor Vergata hospital) to investigate the related working risk factors for SARS-CoV-2 infection, before the beginning of the vaccination programmes commenced in 2021.

## 2. Materials and Methods

This study obtained the approval of the local ethics committee. In our survey, we retrospectively evaluated clinical and serological data of 918 HCWs (all nurses) who underwent occupational COVID-19 screening at the occupational medicine service of a Rome COVID hospital (Tor Vergata hospital) from 1 March 2020 to 31 December 2020. According to working status, we separated the study population in these groups: (1) night shift workers (NSW) (*n* = 445) who perform two-to-seven nights per month followed by two days off, and (2) daytime-workers (*n* = 471) who never work at night. Moreover, we combined participants according to their setting of employment in two different occupational risk group: high-risk departments (infectious disease, emergency setting, pneumology and internal medicine) vs. low-risk departments (all remaining including the no COVID-19 Intensive Care Unit). The hospital screening for Sars-Cov-2 infection was widely recommended but not compulsory. Nevertheless, the adherence of hospital nurses to the screening was complete. Participants were periodically screened (every 15–30 days) undergoing a SARS-CoV-2 real-time reverse transcriptase-polymerase chain reaction nasopharyngeal swab tests (AllpexTM 2019-nCOV Assay). Sensitivity and specificity of the test were estimated to be 74% (95% C.I. 68–80%) and 99.7% (95% C.I. 99.5–99.9%), respectively. Moreover, all subjects underwent periodical serological evaluation (every 30 days) for anti-nucleocapside SARS-CoV-2 IgG using the LIAISON^®^ SARS-CoV-2 Ag assay that is a chemiluminescence sandwich-immunoassay (CLIA)-based technology for the quantitative determination of nucleocapsid antigen protein from SARS-CoV-2 samples. Declared sensitivity and specificity of the test were 98.6% (95% C.I. 92.5–99.7%) and 99.5% (95% C.I. 97.4–99.9%), respectively. Participants tested positive for Sars-Cov-2 infection in both SARS-CoV-2 PCR swabs, and SARS-CoV-2 IgG assay (*n* = 21) was considered only once in results. Notably, we did not observe any cases of re-infection within the study period. During the first visit of the periodic screening, the Occupational Medicine Service of Tor Vergata Policlinic, Rome, collected participant’s data by a clinical interview. We also collected clinical and demographic information such as sex, age, smoking habit and body mass index (BMI).

### Statistical Analysis

Quantitative data were reported as mean ± SD (standard deviation). Categorical variables were indicated as number (percentage) of participants. As mentioned before, the aim of this research was to develop a retrospective investigation for a descriptive purpose. We used univariate logistic regression model to estimate odds ratios for SARS-CoV-2 positive test. We included in the model the following factors: age, sex, smoking status, night shift status and risk classification. Variables were selected only if related to the major outcome in univariate analysis and were considered in the final model. All *p*-values were two-tailed, and we set the significance level at 5%. All analyses were carried out with the 19.0 version of SPSS for Windows (IBM Corp., Armonk, NY, USA).

## 3. Results

**Figure 1:**
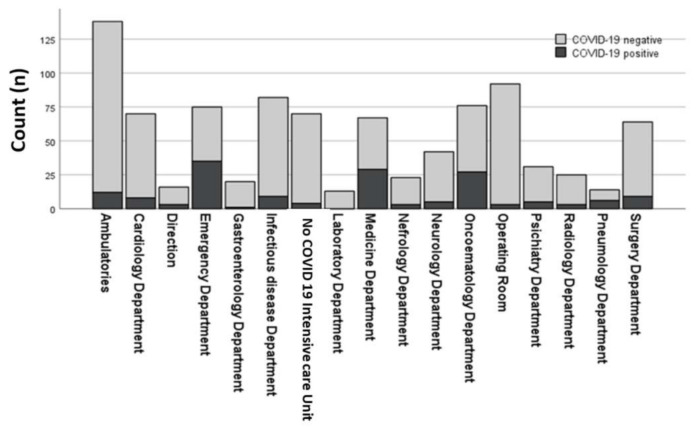
Number of COVID-19 nurses (positive and negative) in study population divided according to hospital departments.

We found 162 cases of SARS-CoV-2 infection (*n* = 143 nasopharyngeal swab and *n* =19 IgG-positive); 157 individuals manifested asymptomatic or mild-to-moderate symptoms, whereas 5 individuals developed severe clinical course. The prevalence rate was 17.6% and the incidence rate was 11.2 COVID-19 cases per 1000 individuals for one month. Regarding the main studied variables, most SARS-CoV-2-infected nurses were NSWs with higher BMI and lower mean age. Compared with never and former smokers, the rate of current smokers was higher in the COVID-19 operators, whereas there was no difference between the two groups related to the gender. Notably, the risk of SARS-CoV-2 infection was statistically related to the clinical care environment. As shown in Figure 1, most of infected operators worked in Internal Medicine and Emergency Departments. Compared to employers in low-risk Departments, nurses working in Infectious Disease, Pulmonary, Internal Medicine, and Emergency Department had higher risk for COVID-19 infection (*p* < 0.001, Table 1). Interestingly, compared with those working in other low risk Departments, only nurses operating in the Oncohematology Department showed a particularly higher level of SARS-CoV-2 infection.

In a regression model, age, night shift work, smoking status and working in high risk settings were statistically related to hospital SARS-CoV-2 infection, independently of other study variables, whereas BMI was not significantly related (Table 2). In particular, NSW was related to a remarkable risk of SARS-CoV-2 infection among HCWs, with a 2.056 estimated odds ratio (95% C.I. 1.320–2.300). Notably, working in departments at high risk of SARS-CoV-2 infection was statistically related to the study outcome, also afterwards adjusting for the main covariates (OR 1.607, 95% C.I. 1.036–2.493).

## 4. Discussion

In the present study, carried out during pre-vaccine period, we found a rate of SARS-CoV-2 infection in HCWs equal to 17.6%, which is higher than the value estimated in the general Italian population. A possible explanation is related to the extreme variability of infection rate found in different studies. Many factors may contribute such as sensitivity and specificity of commercial serological tests used in the different analysis, heterogeneity regarding the statistical power of the analysis and not least, the period of the studies that corresponded to the different waves of the virus spread in different regions and countries. Moreover, a large part of those studies was completed at the end of the first wave of epidemic; therefore, they did not include the significant number of operators who became affected at the end of 2020. These points make difficult a comparison between our results and those reported in previous studies. It remains unclear whether this discrepancy derives also from some form of underestimation due to the presence of asymptomatic subjects, or simply due to a large number of undiagnosed subjects in general population. However, our finding is consistent with WHO records [2], reporting that 14% of all COVID-19 cases involved HCWs, whereas they represent only the 2–3% of the general population in high-middle income countries. Overall, it results in a seven-fold higher rate of contagion than the general population.

Notably, we reported that night shift work was significantly correlated to SARS-CoV-2 infection risk. This finding added a new negative consequence for the health of the shift workers and confirms our preliminary report, as well as other observations in the Italian population [12,15]. This association could be the result of a strong workload during prolonged night shifts that might lead to a decrease in attention in the use of protective devices. However, whether the circadian misalignment and the disruption of rhythmic expression of clock genes (Per2, Rev-ERBα, and BMAL1) [16] in night shift workers could lead to a major risk of SARS-CoV-2 infection and development of a more severe disease remains to be determined.

The association between SARS-CoV-2 infection and high-risk health care setting is not surprising and consistent with previous reports. In recent findings from USA and the UK, the risk of infection for frontline HCWs was estimated to be 3.4-fold higher than the general population [3]. The appropriate training of hospital nurses apparently contrasts with the high rate of contagion detected in the study. However, at the beginning of COVID-19 pandemic, inadequate personal protection due to a lack of virus understanding, protracted exposure to a large number of infected patients as well as the lack of personal protective equipment and psychological pressure were the leading causes of HCW contagion [17]. Consistently, those factors were present in our population.

While it is known that the evolution of COVID-19 is more serious in patients with pulmonary chronic diseases such as COPD [18], and active or former smoking are evidently related with severe COVID-19 [19], few studies explored the correlation between smoking habit and the risk of SARS-CoV-2 infection in HCWs [20]. In this regard, our finding clearly indicates that current or former smoking behaviour is related to increased SARS-CoV-2 infection risk compared with non-smokers (OR = 1.603, 95%CI 1.080–2.378, *p* = 0.019).

Obesity is recognized as a remarkable risk factor for COVID-19 [12,21,22], and high expression of ACE2 may be related to an elevated amount of SARS-CoV-2 infection of the respiratory tract in overweight and/or obese people [23]. Moreover, obesity can restrict ventilation by hampering diaphragm shifting and impair immune responses to viral infection, leading to a severe clinical outcome. Unexpectedly, in our study we did not find any clear association between COVID-19 risk and BMI. However, it is conceivable that the lack of relation occurs because the majority of study participants had a BMI within normal range. Furthermore, our results pointed out the higher incidence of SARS-CoV-2 infection among young nurses. This could be related to a minor experience in protective devices use, as well as greater social activity outside the hospital.

Our study has some limitations. First, we used a retrospective analysis, and not a prospective approach to dates. Second, although contagion outside the work environment is a common method of SARS-CoV-2 diffusion, we did not collect information about possible infection outside the hospital. Moreover, the number of participants was unbalanced as well as the mean age was different between the groups. Furthermore, we are unable to provide details regarding the nurse’s mobility during the study period. In fact, it is possible, albeit rarely, that some nurses were moved from one department to another due to emergencies. This limitation may have certainly influenced our results.

However, several characteristics of this study are notable. Firstly, as far as we know, this is the first study reporting a significant association between area of employment and the risk of contagion among a highly screened hospital population. These findings are of great importance from both a preventive and insurance point of view since in Italy, COVID-19 infections of health workers are specifically compensated, even in the absence of a known exposure. Moreover, the study population is highly homogeneous, including exclusively hospital nurses. Nurse occupation involves close contact with patients for a protracted time during the work shift, including the execution of manoeuvres having high risk of diffusion of airborne pathogens, including SARS-CoV-2. Nurses, in fact, have been often involved in the reported cases of occupational contagion and their protection is a major occupational health issue [17,18,24]. Furthermore, our data are an average of COVID-19 infection cases over a long period because they were collected during the two pandemic waves which occurred in Italy from 1 March 2020 to 31 December 2020.

## 5. Conclusions

Our findings, resulting from a COVID-19 pre-vaccine period, clearly indicate that the protection of HCWs represents a public health priority as the inability to preserve the health and safety of frontline operators raises a risk of collapse of the health system and may drive the spread of the infection from health care settings to the community.

## Figures and Tables

**Table 1:** Main population characteristic.

Variables	COVID+ (*n* = 162)	COVID- (*n* = 756)
Age (years)	42.7 ± 8.8	47.3 ± 5.8
Sex (male/female)	35/127	184/572
BMI	24.5 ± 3.0	23.9 ± 3.6
Smoking (current or former/never)	54/108	190/566
Night shift workers (yes/no)	117/43	328/428
Working department (high risk/low risk)	76/86	151/605

**Table 2:** Univariate and multivariate analyses of the study outcome.

	Univariate OR	Lower-Upper Bound	Multivariate OR	Lower-Upper Bound
Age	0.896	0.871–0.892	0.923	0.895–0.952
BMI	1.046	1.000–1.094	1.023	0.975–1.073
Sex (male)	0.857	0.569–1.290	0.864	0.559–1.337
Smoking (current/former)	1.489	1.033–2.147	1.603	1.080–2.378
Night shift work	3.550	2.433–5.181	2.056	1.320–3.200
High risk health care setting	3.541	2.479–5.057	1.607	1.036–2.593
